# The Effect of Substitution of Palm Fat with Linseed Oil on the Lipid Peroxidation, Antioxidative Capacity and Intestinal Morphology in Rabbits (*Oryctolagus cuniculus*)

**DOI:** 10.3390/ani9100830

**Published:** 2019-10-19

**Authors:** Tina Trebušak, Milka Vrecl Fazarinc, Janez Salobir, Tatjana Pirman

**Affiliations:** 1 Department of Animal Science, Biotechnical Faculty, University of Ljubljana, Groblje 3, 1230 Domžale, Slovenia; tina.trebusak@gmail.com (T.T.); Janez.salobir@bf.uni-lj.si (J.S.); 2 Institute of Preclinical Sciences, Veterinary Faculty, University of Ljubljana, Gerbičeva 60, 1000 Ljubljana, Slovenia; milka.vreclfazarinc@vf.uni-lj.si

**Keywords:** polyunsaturated fatty acids, malondialdehyde, antioxidative capacity, histology

## Abstract

**Simple Summary:**

By changing the source of fat in the diet of animals, the fat content of animal products, such as meat, could be improved. The addition of linseed oil, with a high proportion of polyunsaturated fatty acids, especially n-3 α-linolenic acid, is often used in animal nutrition. Rabbit diet manipulation is effective in increasing or changing the level of fatty acids such that meat could become a functional food for humans. Since polyunsaturated fatty acids are susceptible on oxidation, an effect on animal health could arise. Therefore, we conducted a trial replacing palm fat (saturated fatty acids) with linseed oil (polyunsaturated fatty acids) in the rabbit diet to compere the oxidative status and histology of intestinal tissues. There was no significant effect in the substitution of linseed oil on the malondialdehyde concentration in urine and plasma, antioxidative capacity of water soluble or lipid soluble antioxidants, or intestinal morphology. Therefore, with an appropriate dietary strategy, the content of rabbit meat can be improved without reducing animal welfare.

**Abstract:**

This experiment was conducted to investigate the effect of different dietary fatty acids (saturated or polyunsaturated fatty acids) supplementation on the oxidative status and intestinal morphology of adult rabbits (*Oryctolagus cuniculus*). Twenty-four “slovenska kunka” rabbits were randomly assigned to two different dietary treatments (12 rabbits per treatment) and fed the experimental diets between 80 and 102 days of age. The palm fat (PALM) diet with 6% palm fat and linseed (LINSEED) diets with 6% linseed oil were used. To evaluate the oxidative status of rabbits, the malondialdehyde concentration in urine and plasma and concentration of water and lipid soluble antioxidants in plasma were measured. The antioxidative capacity of the gastrointestinal tract was evaluated by measuring concentration of water and lipid soluble antioxidants in tissues and contents of the intestine. The histological structure of the small intestine and caecum was analyzed via histomorphometric analysis. No significant differences were found in either of those parameters. In summary, rabbits were exposed to high levels of polyunsaturated fatty acids with a high predisposition to oxidation, but their health and welfare were not endangered.

## 1. Introduction

Polyunsaturated fatty acids (PUFA) play an important role in human nutrition, especially n-3 PUFA, which have several beneficial effects on human health, and has been documented by numerous studies [1,2]. Recent recommendations for human diets suggest increasing n-3 consumption and decreasing the ratio of n-6 to n-3 PUFA to 5/1 or even lower [3]; therefore, the interest in obtaining animal product enriched with n-3 PUFA has increased, and this could be done with an appropriate dietary strategy. However, the addition of a higher amount of PUFA in a rabbit diet could have a negative effect on the production and health of the rabbits [4,5,6] since rabbits are very sensitive to nutrition alteration. On the other hand, the digestibility of fats depends of the saturation of the fatty acids [7]. Fat addition to the diet also has a limitation of only 30 g of fat/kg diet due to the pelleting process and possible destruction of microbial balance due to nondigested fat in the intestine [6]. In growing and fattening rabbits, fat supplementation may produce a favorable change in the fatty acid profile and the nutrition value of rabbit meat [8]. Linseed or linseed oil is a suitable and frequently used plant source of n-3 PUFA due to its high α-linolenic acid (C18:3 n-3) content, the consequence of which is improved fatty acid composition of the rabbit meat [9,10]. Unfortunately, PUFA easily undergoes peroxidative damage, so the higher content of PUFA in meat leads to a higher susceptibility of lipid oxidation (formation of free radicals and aldehydes, like malondialdehyde) during the storage and cooking of rabbit meat [10,11], as well as other sources of meat [12,13] if the protection with an addition of antioxidants in the diet isn’t high enough [13,14,15,16,17].

Triglycerides ingested by rabbits in the diet are submitted to complex process of digestion and absorption. The enzymatic hydrolysis of triglycerides leads to the separation of glycerol and free fatty acids formatting microscopic micelles, which move to the microvilli of the duodenum and jejunum, which absorb them [18]. Dietary manipulation, even when diets reach the nutrition requirements in terms of the content of energy, protein and other nutrients, can modify the histological structure of small intestine, leading to alteration in the absorptive surface area of villus cells [19]. Feeding the high-fat diet produce an increase in the height of the villus, on the contrary, the changes in crypth depth not occurred [20]. The most important dietary macronutrient for developing of the morphological features of the villi are crude proteins, since the villus height in the duodenum and ileum were lower by the chickens fed low-crude protein, but not by ingestion of low-crude fat diet, at the same level of metabolizable energy in the diet [19].

The aim of the present study was to assess changes in oxidative status and the histological alterations of the intestinal villi and crypt of the rabbits, if the saturated fatty acids (SFA) in the diet were replaced with PUFA. Replacing SFA with PUFA in the diet of rabbits, the functional food for humans (meat) can be produced, but what influence might this have on the health status of rabbits and the histology of intestinal tissues?

## 2. Materials and Methods

### 2.1. Diets

Two diets were prepared, a palm fat (PALM) diet with 6% palm fat and linseed (LINSEED) diet with 6% linseed oil, which was the only difference in the ingredients of the diets (Table 1). Linseed oil contains more than 65% PUFA and is known to increase dietary oxidative stress. In our previous studies on poultry and pig was proven, that inclusion 5–7.5% of linseed oil in a diet increase fat oxidation products and with that the oxidative stress [13,21,22,23]. Under such circumstances, the recommendations for vitamin E did not seem to be enough to alleviate the negative effects of a large amount of n-3 PUFA in diets. Palm fat is a fat with very low PUFA content (2% linoleic acid) and thus does not increase nutritive oxidative stress, so it was used as a control.

During the experiment, both diet samples were taken for the purpose of proximate analysis and for the determination of fatty acid composition (Table 2). Proximate analysis was determined in our laboratory by standard procedures [24]: dry matter on oven drying at 95–100 °C (AOAC Official method 934.01), crude protein by copper catalyst Kjeldahl method (AOAC Official method 984.13), crude fat or ether extract by extraction in petroleum ether (AOAC Official method 920.39), crude fiber by fritted glass crucible method (AOAC Official method 978.10), and crude ash (AOAC Official method 942.05). The fatty acid composition of diets were analyzed using a gas chromatographic method after the *in-situ* transesterification of lipids. Each sample was analyzed in duplicate. Methyl esters of fatty acids were prepared according to the procedure of Park and Goins [25].

### 2.2. Animals

All procedures with animals were performed according to current legislation on animal experimentation in Slovenia and the animal experiment was permitted by the Animal welfare body at the Department of Animal Science according to VURS (Veterinarian administration of Republic of Slovenia) letter No. 34401-55/2007/2. Animals used in this experiment were reared and slaughtered at the Department of Animal Science, Biotechnical Faculty (Ljubljana, Slovenia).

Twenty-four 75-days old SIKA, “slovenska kunka” rabbits (*Oryctologus cuniculus*) were housed individually in two-floor wire cages (45.5 × 37.5 × 30 cm) with free access to drinking water (nipple drinkers) and a pelleted diet. The room temperature was 20 °C with 60% humidity and a light-dark cycle of 12 h light/dark starting at 7:00 a.m. In the adaptation period (5 days), rabbits received commercial pellets *ad libitum*. After the adaptation period, at 80 days of age, rabbits were randomly divided by gender and weight (2580 ± 299 g of body weight) into two groups (n = 12) with different dietary treatments (Table 1) for 22 days. Each day, animals received a weighed daily meal and the residue from the day before was weighed and discarded. Body weights were recorded weekly during the experimental period and just before slaughter. During the experiment, one animal (female from the LINSEED group) had health problems (feet blister) and was eliminated from the experiment. At 102 days of age, rabbits were stunned by electricity (70 V, 50 Hz) and slaughtered by cutting the carotid arteries and jugular veins, then exsanguinated in accordance with the Slovenian national regulations for commercial slaughtering.

### 2.3. Tissue Sampling

In the last week of the experiment, six animals of each group were housed for three days in metabolic cages where cumulative urine samples were collected for 48 h, registering the amount of collected urine for each animal, and homogenized aliquot was transferred into 2 mL Eppendorf tubes and stored at −80 °C until analysis. Blood samples were collected at slaughter into 6 mL evacuated tubes containing EDTAK_2_ anticoagulant (367864, BD-Plymouth, UK). Plasma was separated by centrifugation (1000× *g* for 15 min at 4 °C), transferred into Eppendorf tubes, and stored at −80 °C until analysis.

After slaughter, the intestinal tract was removed and divided into duodenum, jejunum, ileum, and ceacum. Tissues samples of the small intestine and caecum were taken on the following: duodenum, the final part of descending duodenum (*pars descendens duodeni*) before the caudal duodenal flexure (*flexura duodeni caudalis*); jejunum, middle part of the jejunum; ileum, part of ileum before its extended part (*ampulla ilei*); caecum, part of the caecum between the first and second gyrus (*gyrus primus* and *gyrus secundus caeci*). Samples were cleaned and fixed in 5% buffered formalin solution until the analyses were performed.

The digestive content of the small intestine was collected by finger pressure, put in the Eppendorf tubes, and stored at −80 °C until an analysis of antioxidative capacity in water and in lipid soluble antioxidants were performed. A 3 cm length of small intestine tissues was taken from the middle section for the antioxidative capacity analysis and stored at −80 °C until the analyses were performed.

### 2.4. Malondialdehyde (MDA) Determination

Concentration of MDA in urine and plasma were measured according to the methodology of Wong et al. [26] modified by Chirico et al. [27] and Fukunaga et al. [28], using HPLC, and the quantification of MDA was performed using external standard (TEP, 5 point calibration curve). The procedure for the MDA determination in plasma was already described in Trebušak, et al. [29]. Briefly, for urine MDA determination, 100 μL of urine was mixed with 100 μL of 0.44 M H_3_PO_4_ and 10 μL of 0.2% BHT in the Eppendorf microcentrifuge tubes and left for 15 min before adding 300 μL ethanol and then centrifuging (15,000× *g*, for 15 min at 4 °C). The supernatant (350 μL) was mixed with 1.5 mL of 0.44 M H_3_PO_4_, 0.5 mL of 0.6% TBA, and 0.9 mL of Milli Q deionized water in a tube with stopper and heated at 90 °C for 60 min. After cooling, the samples were filtered through Millipore filters into auto sampler vials. To determine urine and plasma MDA, a Waters Alliance 2690 (Waters, Milford, MA, USA) equipped with Waters 474 scanning fluorescence detector was used. For the purpose of separation, a reversed-phase HPLC chromatography column (HyperClone 5μm ODS (C_18_) 120 A, 4.6 mm × 150 mm × 5 μm; Phenomenex Inc., Torrance, CA, USA) and C_18_ ODS guard column (4 mm × 3 mm; Phenomenex Inc.) was used. The mobile phase consisted of 65% 50 mmol/L KH_2_PO_4_ buffer (pH 6.9) and 35% methanol. The mobile phase flow rate was 1.0 mL/min. The results of the analysis were evaluated using the Millenium^32^ Chromatography Manager (Waters, Milford, MA, USA) program.

### 2.5. Antioxidative Capacity of Plasma and in Tissue and Content of Small Intestine

The antioxidative capacity of the lipid soluble (ACL) and water soluble (ACW) compounds in blood plasma, as well as in tissue and content of the small intestine, was measured using the photochemiluminiscence method by PhotoChem^®^ (Analytik Jena, Jena, Germany) and presented as Trolox equivalents (ACL) or ascorbic acid equivalents (ACW). The procedure for plasma was as follows: 200 μL of plasma and 200 μL of methanol (Sigma-Aldrich Chemie GmbH, Munich, Germany) were mixed in a vortex and centrifuged (15,000× *g*, 10 min, 4 °C). The supernatants were analyzed according to an ACL-Kit protocol (Analytik Jena, Jena, Germany). For the water soluble antioxidants, the sample of plasma was analyzed according to ACW-Kit protocol (Analytic Jena, Jena, Germany). The tissue sample (0.5 g) was homogenized in a Potter, ground-glass homogenizer with 3 mL of a (1:1, *v/v*) mixture of NaCl solution (5%, *w/v*) and ethanol. 3 mL of hexane was added, followed by homogenization and centrifugation (15,000× *g*, 10 min, 4 °C). The hexane layer was collected and the extraction in hexane was repeated. The hexane extract was dried under nitrogen and the residue was dissolved in methanol and analyzed according to ACL-Kit protocol (Analytik Jena, Jena, Germany). The remaining aqueous layer was also collected, and the water soluble antioxidants were analyzed according to ACW-Kit protocol (Analytic Jena, Jena, Germany). Content of the small intestine was lyophilized and homogenized prior to the analytical procedure, the same as for the tissue samples.

### 2.6. Histologic Measurements

Parts of the small intestine (duodenum, jejunum and ileum) and caecum fixed in 5% buffered formalin solution were embedded in paraffin using a standard procedure. Subsequently, an evenly spaced series of histologic section (50 μm intersection interval) were cut at 5 μm and stained with hematoxylin and eosin (H&E).

Histomorphometric analysis was performed on H&E-stained tissue sections using a Nikon Microphot FXA microscope equipped with a DS-Fi1 camera and the Imaging Software NIS Elements D.32 (Nikon instruments Europe B.V., Badhoevedorp, The Netherlands).

**Figure 1:**
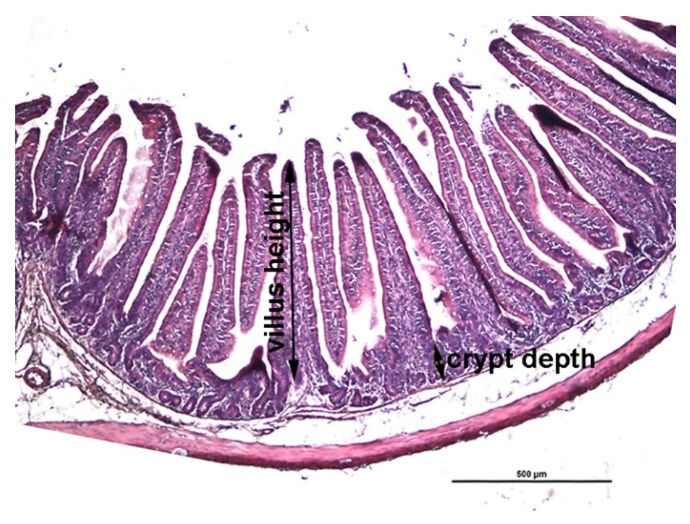
A representative histological cross section of jejunum. Villus height was measured from the tip of the villus to the crypt-villus junction and the crypt depth was measured from the crypt-villus junction to the base of the crypt as indicated. H&E staining; scale bar 500 µm.

Villus height was measured from the tip to the crypt-villus junction and the crypt depth measured from the crypt-villus junction to the base as illustrated in Figure 1.

### 2.7. Statistical Analysis

The data were analyzed by the Student *t*-test in an SAS/STAT module (SAS Institute, Cary, NC, USA), taking into consideration the diet as the only main effect. Significance was considered established at *p* < 0.05.

## 3. Results

### 3.1. Ingredients and Chemical Composition of the Diets

Chemical composition of the diets was similar, but only determination of crude fat showed a slightly lower value in the LINSEED diet (Table 2), even that the amount of the addition of the fat in the diets was at the same level with 1% of rapeseed oil and 6% of palm fat or linseed oil (Table 1). The difference appeared in the content of different fatty acids in the diets (Table 2). Within the LINSEED diet, the level of saturated fatty acid (SFA) was lower and the levels of monounsaturated fatty acids (MUFA) and polyunsaturated fatty acids (PUFA) were higher as compared to PALM diet.

### 3.2. Oxidative Status of Rabbits

Upon completion of the study, average growth rate did not differ significantly (*p* > 0.05) among groups, indicating that diets had no effect on the growth rate of rabbits. Similar results were obtained in diet intake and diet conversion rate (Table 3).

The MDA concentration was similar in both groups in urine and in plasma (Table 4). There was also no significant difference (*p* > 0.05) in the antioxidative capacity of the lipid (ACL) or the water (ACW) soluble compound in the small intestinal tissue and the content of the small intestine, nor in the blood plasma, with the tendency of lower values of ACL and higher of ACW in the LINSEED group, except in plasma (Table 5).

### 3.3. Intestinal Morphology

**Figure 2:**
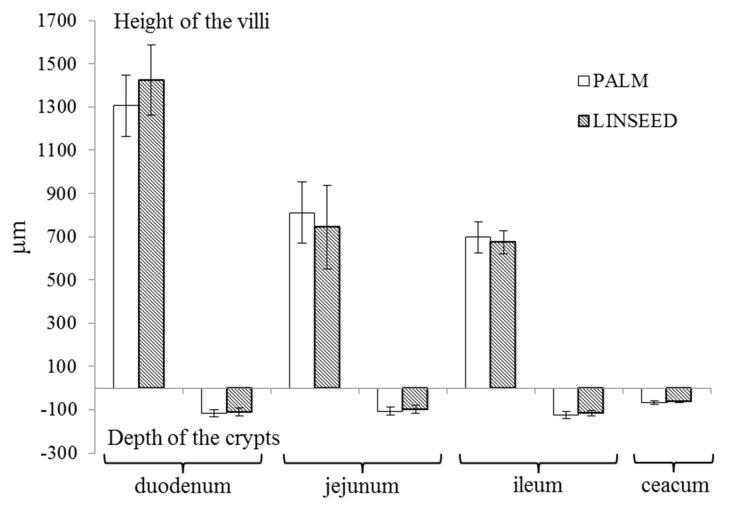
Height of the villi and depth of the crypts in individual parts of the intestine. On the abscissa are different parts of the small intestine: duodenum, jejunum, ileum, and ceacum, on the ordinate is the length in µm, in positive way the height of v the villi and in negative way the depth of the crypt, PALM: 6% palm fat in a diet and LINSEED: 6% linseed oil in a diet.

Our results also showed that linseed oil diet, high on n-3 PUFA, did not significantly affect the histological structure of the small intestine and caecum. The ratio between the height of the villi and depth of the crypt decreasing along intestinal tissues, from 11.3:1 and 12.7:1 in the duodenum to 5.7:1 and 5.8:1 in the ileum, in the PALM and LINSEED groups, respectively (Figure 2).

## 4. Discussion

### 4.1. Oxidative Stress and Antioxidant Capacity

Our results show a similar growth rate and diet conversion rate (Table 3), with a tendency of better results in the LINSEED group, which is in accordance with a report of Maertens, et al. [7] that revealed better digestibility of unsaturated FA compared to saturated FA, but not with Rodríguez, et al. [30], who detected slightly reduced growth of rabbits after the addition of fish oil in a diet, but with younger rabbits. In general, a linseed diet contains a higher proportion of PUFA and MUFA (Table 2), leading to a higher proportion of PUFA due to a lower proportion of SFA in the meat [10], which leads to a higher oxidation in meat [10] and some other tissues [29], but did not affect the MDA concentration of the urine and plasma (Table 4). Oxidation of lipids or fatty acids can be prevented or reduced by the use of appropriate dietary strategies, among which is the most common use of fat soluble antioxidant, vitamin E. The content of vitamin E in the diet of our experiment was apparently sufficient to prevent the oxidation, since the differences in MDA concentration in urine or plasma were not detected. The oxidation of the rabbits was not increased after the addition of dietary flax seed with a high PUFA content [31]. Good oxidation stability of animal diet or linseed oil can also be one of the reasons, which have been proven in subsequent research on the n-3 PUFA enriched feed for laying hens (6% added linseed oil) in our lab (unpublished observation). According to Tres, et al. [17] increasing vegetable fat (up to 30 g/kg) in the diet for rabbits increased susceptibility to oxidation, which was reflected in an increased content of lipid hydroperoxide in plasma. With increased vegetable fat content in the diet, MDA concentration in the meat increased, while MDA concentration in plasma was below the detection limit (13 μg MDA/L plasma) [17]. On the contrary, in pigs and chickens, the higher consumption of PUFA caused lipid oxidation in the plasma. Rezar, et al. [32] reported that an addition of linseed oil (50 g/kg) in a pig diet caused a two-fold higher MDA concentration in the plasma compared to control. Moreover, an addition of 75 g of linseed oil per kg of chicken diet increased the plasma MDA concentration five-fold [13].

Furthermore, diet in our study also had no effect on the antioxidant capacity of the lipid or water soluble antioxidants in the tissue or content of the small intestine, nor in plasma (Table 5). There was some tendency of lower value of antioxidative capacity of lipid soluble antioxidants (ACL) in the content of the small intestine in the LINSEED group, which could mean more efficient absorption of antioxidants or increased consumption of antioxidants to protect the organism against oxidative stress. The plasma ACL values (PALM vs. LINSEED) confirmed used antioxidants to protect oxidation in the plasma, since the plasma MDA content (Table 4) was not affected. In the present study there were no specially added antioxidants or compounds with antioxidant properties, and it was expected that there would be some differences at the ACW or ACL level, between different dietary treatment. In some trials in which the antioxidants were included, the differences in plasma or serum ACW and ACL between the groups was detected [33,34], but in some trials the addition of antioxidants had no effect [23], or potential antioxidants (hops) even decreased the ACL level in plasma (Rezar, not published). There could be many reasons for this discrepancy in the results: different animal species (rabbits vs. poultry, horses, rats, piglets), endogenous antioxidants could be counteracting the presence of the exogenous antioxidants [35], low absorption, strong homeostasis, fast excretion of polyphenols/antioxidants, or fast metabolism [36]. The level of addition of antioxidants/potential antioxidants, or no extra addition and the level of unsaturated fatty acids, or any other potential oxidants, could be yet other reasons.

### 4.2. Histology

Literature examining the histological structure of the digestive tract of adult rabbits is sparse, which is probably due to the fact that the most critical period of rabbits is during weaning and the first 10 to 15 days after weaning [37]. Makovicky, et al. [38] showed that villus height significantly increased with rabbit age. In addition, the anatomical development of rabbit’s digestive tract is stabilized from nine weeks of age, with the exception of the caecal appendix, which grows until 11 weeks of age [39]. In the first postnatal week, major changes in the morphological structure of the gastrointestinal tract occur, which can be seen in the shape of the villi and in the ratio between the height and width of the villi. During subsequent weeks, long and thin villi (finger-shaped) become broader and longer (tongue-shaped) resulting in changed height to width ratio [40]. For example, in rabbits aged 28 and 49 days, the average duodenal villi height was 746 and 940 μm, respectively. Moreover, from two weeks until seven weeks of age, the crypts deepened from 50 μm to 180 μm [41]. Our results showed that the height of the villi decreased towards the distal part of the small intestine, which agrees with results of Incharoen, et al. [19], while the crypt depth was similar in all three parts of the small intestine and was approximately twice as deep as in the caecum. A similar trend of the decreasing the villi high, and no differences in crypt depth in the jejunum and ileum mucosa, with no significant differences, regarding the digestible energy level or protein level of diet, were found by Tazzoli, et al. [42]. Addition of inulin in a rabbit diet increase crypt depth and health of gut [43], supplementation of rabbit diet with whey powder increase gut health, but addition of citric acid, had no positive effect [44]. Also, an addition of 0.4% glutamine in a rabbit diet had a positive effect on intestinal villus high, but depended on the age of animals, the addition of arginine, and arginine and glutamine did not have the effect [45].

Compared to Gallois, et al. [41], results of the duodenum and the villi length in our experiment were longer, and crypt depth were slightly shallower, which leads us to the belief that high incorporation of PUFA in a diet has no detrimental effect on intestinal morphology of rabbits. Oso, et al. [46] show no effect of various additives (probiotics and prebiotics) on ileal morphology, with some smaller villi length, but similar crypt depth to our results. Incharoen, et al. [19] found out that the level of crude protein in the diet is more important for the development of the morphology features of the villi in the intestine as compared to the level of fat in the diet. Based on our results, we can also say that the addition of different types of fat does not affect the histological structure of the digestive tract, which is probably more dependent also on the fiber and particle size of the feed. Desantis, et al. [47] reported that rabbits fed with more finely milled particles (2 mm) in feed had irregularly shaped and significantly longer villi in the duodenum compared to rabbits, of which particles in the feed were roughly ground (8 mm). Beside the longer villi, rabbits fed with finely milled feed also had deeper crypts in the duodenum and caecum, and were significantly deeper in the colon [47]; short intensive feed restriction increased villi height and crypt depth [48].)

Furthermore, the ratio between the height of the villi and depth of the crypt in different parts of the small intestine decreased in the direction of the duodenum to ileum. The ratio in PALM and the LINSEED group were 11.3:1 and 12.7:1 in the duodenum, 7.8:1 in jejunum, and 5.7:1 and 5.8:1 in ileum, respectively. This ratio is an indicator of the digestive capacity of the small intestine. A higher ratio signifies the higher rate of digestion and absorption [49]. De Olivera, et al. [50] evaluated the effect of feed restriction on intestinal mucosa of growing rabbits and found that restriction resulted in reduced villi height and crypt depth, with more expressed differences in the lower part of the small intestine due to a higher nutrient digestibility in the initial part of the small intestine. There could also be age related declines in mucosal surface area and some absorption differences regarding diet composition [51].

## 5. Conclusions

The results of our study demonstrate that substitution of linseed oil, with a high amount of PUFA, α-linolenic acid (n-3), to palm fat (SFA), in a rabbit diet, had no significant effect on oxidative status or intestinal morphology, or on the health of adult rabbits. This enables the addition of n-3 PUFA to the rabbit diet and production of functional food for human consumption enriched with n-3 PUFA without adverse effects on the health and welfare of the animals.

## Figures and Tables

**Table 1:** Ingredients of the diets (g/kg).

Ingredients	PALM	LINSEED
Alfalfa	458.3	458.3
Barley	130.0	130.0
Sunflower meal	210.0	210.0
Hay meal	100.0	100.0
Rapeseed oil	10.0	10.0
Palm fat	60.0	
Linseed oil		60.0
Methionine	0.5	0.5
Lysine	2.0	2.0
Vitamin-mineral mix ^1^	5.0	5.0
Lignobond ^2^	20.0	20.0
Salt	4.2	4.2

PALM: 6% palm fat in a diet; LINSEED: 6% linseed oil in a diet. ^1^ Vitamin-mineral mix (0.5%) (mg/kg vitamin-mineral mixture): vitamin A (10 mg), vitamin D3 (2 mg), vitamin E (60 mg), vitamin K3 (4 mg), vitamin B1 (2 mg), vitamin B2 (7.5 mg), vitamin B3 (50 mg), vitamin B5 (20 mg), vitamin B6 (2 mg), vitamin B12 (10 mg), folic acid (5 mg), biotin (5 mg), choline (333 mg), Fe (156 mg), Cu (32 mg), Mn (161 mg), Co (100 mg), I (2.95 mg), Se (15 mg), Zn (125 mg). The amount of wheat bran is 3.9 g/kg diet. ^2^ Pellet binder.

**Table 2:** Chemical composition and fatty acid composition of the diets.

Composition of Diets	PALM	LINSEED
Chemical composition (g/kg)		
Dry matter (DM, g/kg)	933	912
Crude protein	178	179
Crude fat	108	86
Crude fiber	227	228
Crude ash	69	70
Main fatty acids (% of the total fatty acids)		
C12:0	0.20	0.04
C14:0	0.89	0.12
C16:0	35.81	8.00
C18:0	41.73	4.00
∑ C18:1	7.75	23.48
C18:2 n-6	9.01	22.01
C18:3 n-3	2.78	40.23
∑ SFA ^1^	80.08	13.48
∑ MUFA ^2^	8.05	24.14
∑ PUFA ^3^	11.86	62.38
∑ n-3 PUFA	2.85	40.33
∑ n-6 PUFA	9.01	22.05
n-6/n-3 PUFA	3.16	0.55

PALM: 6% palm fat in a diet; LINSEED: 6% linseed oil in a diet. ^1^ Saturated fatty acids. ^2^ Monounsaturated fatty acids. ^3^ Polyunsaturated fatty acids. The calculated value for Ca in a diets was 9.8 g/kg and P 4.2 g/kg.

**Table 3:** Growth performance from different experimental groups (average).

Growth Performance	PALM	LINSEED	SEM	*p*-Value
Growth rate (g/day)	28.8	33.6	5.23	0.213
Diet intake (g/day)	168.3	178.5	10.13	0.463
Feed conversion ratio (g/g) ^1^	6.09	5.66	0.56	0.361

PALM: 6% palm fat in a diet; LINSEED: 6% linseed oil in a diet. ^1^ Calculated as diet intake/growth rate.

**Table 4:** Concentration of malondialdehyde (MDA) in urine and plasma of rabbits from different experimental groups (average).

MDA	PALM	LINSEED	SEM	*p*-Value
Urine (nmol/mL)	8.22	8.77	0.77	0.632
Urine (μmol/48 h) ^1^	2.87	2.63	0.26	0.511
Plasma (nmol/mL)	0.14	0.14	0.01	0.981

PALM: 6% palm fat in a diet; LINSEED: 6% linseed oil in a diet. ^1^ Urine (µmol/48 h): cumulative amount of excreted malondialdehyde in urine in 48 h.

**Table 5:** Antioxidative capacity of the lipid (ACL) and the water (ACW) soluble compound in tissue and content of the small intestine and in blood plasma (average).

Antioxidative Capacity		PALM	LINSEED	SEM	*p*-Value
Tissue	ACL (nmol/g)	20.9	18.9	2.00	0.501
	ACW (μmol/g)	2.81	2.83	0.28	0.970
Content	ACL (nmol/g)	94.0	79.0	5.90	0.089
	ACW (μmol/g)	5.28	6.38	1.67	0.643
Plasma	ACL (nmol/mL)	273.5	253.2	9.85	0.155
	ACW (nmol/mL)	44.5	40.7	5.21	0.612

PALM: 6% palm fat in a diet; LINSEED: 6% linseed oil in a diet.
